# Setting a research agenda to improve community health: An inclusive mixed-methods approach in Northern Uganda

**DOI:** 10.1371/journal.pone.0244249

**Published:** 2021-01-07

**Authors:** Nicholas Dowhaniuk, Susan Ojok, Sarah L. McKune

**Affiliations:** 1 Department of Geography, University of Florida, Gainesville, Florida, United States of America; 2 Department of Environmental and Global Health, University of Florida, Gainesville, Florida, United States of America; 3 Tropical Conservation and Development Program, University of Florida, Gainesville, Florida, United States of America; 4 Uganda Women's Action Program, Gulu, Uganda; 5 African Studies Program, University of Florida, Gainesville, Florida, United States of America; Helen Keller International, UNITED KINGDOM

## Abstract

**Background:**

The United Nations Sustainable Development Goals stress the importance of equitable partnerships in research and practice that integrate grass-roots knowledge, leadership, and expertise. However, priorities for health research in low-and-middle income countries are set almost exclusively by external parties and priorities, while end-users remain "researched on" not "researched with". This paper presents the first stage of a Community-Based Participatory Research-inspired project to engage communities and public-health end-users in setting a research agenda to improve health in their community.

**Methods:**

Photovoice was used in Kuc, Gulu District, Uganda to engage community members in the selection of a research topic for future public health research and intervention. Alcohol-Use Disorders emerged from this process the health issue that most negatively impacts the community. Following identification of this issue, a cross-sectional survey was conducted using the Alcohol Use Disorder Identification Test (n = 327) to triangulate Photovoice findings and to estimate the prevalence of Alcohol-Use Disorders in Kuc. Logistic regression was used to test for associations with demographic characteristics and Alcohol-Use Disorders.

**Results:**

Photovoice generated four prominent themes, including alcohol related issues, sanitation and compound cleanliness, water quality and access, and infrastructure. Alcohol-Use Disorders were identified by the community as the most important driver of poor health. Survey results indicated that 23.55% of adults in Kuc had a probable Alcohol Use Disorder, 16.45 percentage points higher than World Health Organization estimates for Uganda.

**Conclusions:**

Community members engaged in the participatory, bottom-up approach offered by the research team to develop a research agenda to improve health in the community. Participants honed in on the under-researched and underfunded topic of Alcohol-Use Disorders. The findings from Photovoice were validated by survey results, thereby solidifying the high prevalence of Alcohol-Use Disorders as the health outcome that will be targeted through future long-term research and partnership.

## 1. Introduction

Public health issues in Low-Middle Income Countries (LMICs) are integral to many of the United Nations Sustainable Development Goals (SDGs) [1], as illustrated by a recent special section devoted to the topic in the official Journal of the American Academy of Nursing, Nursing Outlook [2–5]. Sustainable Development Goal 17.16 outlines the need to "enhance the global partnership for sustainable development, complemented by multi-stakeholder partnerships that mobilize and share knowledge, expertise, technology and financial resources, to support the achievement of the SDGs in all countries, in particular developing countries [6]." However, health and development research remains an area where priorities are set almost exclusively by a researcher's individual interests or by funding organization priorities, while end-users (especially those from marginalized communities) remain "researched on" and not "researched with" [7–11]. Transdisciplinary partnership, multi-stakeholder collaborations, and grass-roots engagement are considered vital to achieving the SDGs [12]. Community-based interventions are an important entry point for primary and preventative health care [13,14], where community leaders, community groups, and village health workers are vital assets in case-detection, management, and prevention of disease and illness, communication of information regarding risk factors, and the reduction of stigma [15–17]. Recent calls from the medical anthropology community have stressed the importance of ground-up, open-ended, community-based research that prioritizes and gives voice to preferences, perceptions and priorities of the community and is adaptive to unanticipated changes in the research agenda, based on community input [18]. Community-Based Participatory Research (CBPR) is a well-established research approach that holds particular promise to improve inclusion of marginalized communities in the processes necessary for progress towards the SDGs [19]. Research that is based on community goals can increase the usefulness and relevance of data collected [20,21], and improve alignment between funding opportunities and community concerns. Inclusive community approaches, including CBPR, are held up as guideposts for ethical health research, yet are often overlooked [22–26].

Community-Based Participatory Research leverages the researcher's theoretical expertise with the invaluable lived experience of community members [27–29]. Community-Based Participatory Research integrates local biopsychosocial models and helps minimize culture-based assumptions that are often inherent in global health research [30], effectively bridging the recognized gap between local and global knowledge [18,31]. Community-Based Participatory Research has increasingly been recognized as an important means of connecting research and practice [32–34]. This method ensures greater community involvement and control in the research process [35], and better identifies and understands the issues of vulnerable and marginalized communities [33].

Photovoice is a participatory method used increasingly in public health research and heralded for its effectiveness in uncovering and empowering community perspectives of health needs and strengths within a community [36,37]. The Photovoice methodology first entails distribution of cameras to community members to document their perspective and life experience, in response to a prompt by the researcher, through photographs. After a designated period of time, the researcher then conducts interviews and focus groups with participants, where the photographs taken by participants are used to facilitate discussion and reflection on participant and community perspectives of the research prompt. Photovoice is an effective method of communicating ideas across culture and language barriers [38–41] that empowers and gives marginalized and vulnerable populations a voice, and effectively identifies a research issue using a participatory framework [42,43]. Photovoice is a method rooted in the Participatory Action Research (PAR) paradigm [44,45], with theory borrowed from feminist theory and documentary photography [37]. Participatory Action Research differs from other qualitative methodologies in that the primary purpose of PAR is to influence social change through an iterative process of knowledge generation, action, and reflection [45]. Photovoice helps develop "critical consciousness", where reflection through group dialogue and participatory action creates awareness and influences Praxis, defined as informed and reflective collective action to address an issue [46–49].

### Research motivation

This article aims to provide documentation and reflection on the first phase of an evolving research process. Having established the importance of *how and on what topic* research is conducted, we present here findings from a bottom-up approach by which community members were engaged in the selection of a research topic for future public health research and intervention. Indeed, in the spirit of PAR, this process was not initiated solely for academic purposes, but represents the first stage of a long-term CBPR-inspired project to investigate a community selected research topic, with the goal of co-developing a community-based intervention or solution to that health need. As a byproduct of this work and its design, two additional research questions are addressed in this article: 1) using Photovoice, what are the major drivers of health within the Kuc community, including both the health issues that are most detrimental and the community assets that drive/could drive positive health-related change, and 2) given past scholarly criticisms of the Photovoice, does triangulation of those findings with survey-based data, in which a larger group of community respondents answer privately about their perception of health problems and drivers of health within the community, validate concerns identified through Photovoice [50]? Through this process, we conclude that long-term research design, goals, and implementation should more affectively allow for and engage stakeholder and beneficiary input. This process will better serve community health needs, goals, and assets, while simultaneously fostering critical consciousness and community-level engagement.

## 2. Materials and methods

### 2.1.Study area

Kuc (a pseudonym for a village located in Aswa County, Gulu District, Uganda) is located in Northern Uganda in East Africa. The county was the site of a humanitarian crisis during the insurrection of the Lord's Resistance Army in northern Uganda from 1986 to 2006. During the conflict, more than ninety-five percent of the population of Gulu District was forced into Internally Displaced Persons (IDP) camps [51]. The war left a significant mark on human livelihoods in the region, including lasting negative effects on health infrastructure, disease, and disability [52]. Significant investment and concerted efforts in response to the conflict among domestic and international government and non-governmental organizations has resulted in Gulu District being one of the top performing districts in health [53].

While village level census data are not available for Kuc, the 2014 Uganda Census [54] provides details for Aswa County. In 2014, Aswa County had a population of 125,307 people, 58% of whom were 17 years of age or less. Approximately 39% of the population over 18 years of age was illiterate at that time, and 82.8% lived in temporary dwelling units. Access to community services was varied, with 86.8% of the population within 5km of the nearest public school, 34.7% within 5km of the nearest secondary school, and 72.8% within 5km of the nearest health facility. Mosquito net ownership was high (97.7%), while access to water was most commonly drawn from a borehole (45.2%), household sanitation facilities were common (87.1% own a toilet facility), and the population was largely reliant on subsistence farming as a main source of livelihood (87.5%).

### 2.2.Photovoice

#### 2.2.1. Establishment of Community-Advisory Board

Three individuals representing diverse community interests were recruited to a Community Advisory Board (CAB) [55,56]. Identification and recruitment of these board members occurred through informal discussions with community leaders and with community members at community events, with the final selection being made by the research team. Eligibility for CAB selection included having lived in the community since the conflict, being widely connected within and respected by the community, not being a politician, and having a reputation for being responsible and trustworthy. Members of the CAB, which is still a functioning body supporting the ongoing efforts associated with this work, are a head teacher at the primary school, the leader of a local women's organization, and a prominent and well-connected representative of a local non-governmental organization. The CAB represented a voice for the community during the research process [55] to ensure the project was conducted in a culturally sensitive manner. They also provided input, feedback, and verification of the results and interpretation. Finally, they provided important instruction on project direction and served as a general source of community knowledge and counsel.

#### 2.2.2. Participant selection and photography workshop

Following other Photovoice studies [57–59], eight participants were selected through purposive sampling to enable in-depth discussion and gain highly contextualized interview data. A summary of participant demographics is presented in Table 1. Five women and three men were selected, ranging in age from 25–50, and representing various geographic areas of the Kuc community. Members of the CAB were asked to recommend individuals who were informed about community issues, were responsible community members, and would take seriously the responsibility of representing the community, and the research team made the final selection based largely on these recommendations. Community Advisory Board members contacted the participants directly to inquire if they would be willing to participate in the project. If the participants expressed interest, they were recruited to attend a participant meeting where enrollment was finalized. By limiting the sample to eight participants, the research team ensured that each participant would have a camera for two weeks, resulting in two groups of four people collecting photos at a time; this approach was necessary given funding constraints and camera availability. A six-hour training session on photography and photography ethics was conducted at the first meeting, which included obtaining written informed consent from the participants for the photography exercise, individual interviews and the focus discussion. Participants were provided ample time to ask questions. Participants were also trained on how to obtain verbal consent from individuals in a photograph collected by a participant. The theme, "what in your community has a negative or positive impact on your health?", was used as a general prompt for residents to collect photographs that reflect their view of health and health issues. In keeping with Photovoice methods, the theme was specific enough to be directed, but deliberately vague to minimize bias and influence on photographic decisions of participants. Participants collected photographs during December 2018 and January 2019.

**Table 1:** Summary of participant demographic information.

Statistic	Age	Highest Level of Education Attained	Number of Children	Occupation
Range	25–50	Primary 2—Secondary 4	1–7	Farmer, beadmaker, boda boda driver, preacher
Average	39.38	N/A	4.88	N/A

#### 2.2.3. Individual interviews

Photographs were uploaded to a tablet, and each Photovoice participant was asked to review and select his/her five photographs that best represented community health in Kuc. This narrowed down potential topics, allowed for critical reflection, and ensured ample time to discuss individual photographs without diluting discussion due to an abundance of photographs. Semi-structured interviews were conducted, during which the participant shared his/her interpretations of selected photographs and elaborated on his/her perceptions of community health. The study adapted the SHOWeD method—a set of five questions often used in Photovoice to structure individual interviews used to gain insight into the meaning of photographs collected by participants [37,46]—as a guide for the individual interviews (Table 2). The questions were adapted to the local context to ensure there was accurate translation between English and Acholi. Clarifying questions and probes followed each response to inquire further with the participant. The interviewer did not inquire about any specific topics; probing questions were only used after a topic was first mentioned by the participant, and probes were used consistently across topics across participants once the participant provided that level of information–such as a health problem or strength of the community. Individual interviews lasted between forty-five and seventy-five minutes and were conducted in January and February 2019.

**Table 2:** Original SHOWeD method questions and the adapted SHOWeD method questions used in this study.

Question #	SHOWeD Questions	Adapted SHOWeD Questions
1	What do we see here?	What do you see here?
2	What is really happening here?	Why did you take this photo?
3	How does this relate to our lives?	How does this relate to your life and the life of your community
4	Why does this situation, concern, or strength exist?	Why does this situation, concern, or strength exist?
5	What can we do about it?	What can we do about it?

#### 2.2.4. Focus group

All eight Photovoice participants came together in one focus groups to discuss the collection of their selected photographs and the health issues that had been identified in the individual interviews. While the themes that emerged from individual interviews were shared with focus group participants, they were asked the same adapted SHOWeD questions, this time referencing all participants photos. In this way, the focus group served as a space to confirm, deny, and complicate interview results, as well as providing a space for the social interaction between group members where new or additional themes could be generated or existing themes problematized. Interactions between participants can serve as an important mechanism for understanding differences in perceptions across demographic characteristics of participants and geographic location of residence in the village. Additionally, in the spirit of CBPR, the focus group discussion was used to ensure that the topic of most importance identified through the analysis of individual interviews aligned with the topic chosen by the group. This ensured that the most important topic would be pursued in the continuing phases of the CBPR project. The focus group also provided a chance for participants to reflect on health issues and solutions through group dialogue to influence critical consciousness and grass-roots collective action to address an issue. The focus group was conducted at the end of February 2019 and lasted approximately two hours.

#### 2.2.5. Photovoice data analysis

Both the individual interviews and the focus group were conducted using a translator, who worked in real-time to translate questions and responses between Acholi and English. Participants spoke in Acholi, their mother-tongue, necessary to ensure maximum proficiency and comfort when speaking. Translation was provided by a trained research assistant, who worked closely with the research team over the course of the project, was also a native Acholi speaker, but was fluent also in English. Individual interviews and the focus group were recorded and the audio recordings were transcribed by the first author and research assistant, with Acholi responses translated orally to the first author by the translator and directly transcribed into English. The qualitative analysis program, NVIVO 12, was then used to thematically code the interviews using two-cycle coding [60,61]. Holistic coding was used for first-cycle coding, where thematic codes were assigned to the data inductively and segment transcripts into dominant health themes, followed by the use of sub-coding as second-cycle coding to further classify primary codes into common themes nested within primary codes [62,63].

#### 2.2.6. Photovoice data quality and rigor

Data quality and rigor were managed through memoing and member checking [64,65]. Memoing was used to maintain a written account of the first author’s thoughts, perceptions, and observations during all phases of research and to ensure a self-reflexive practice throughout data collection, which is in keeping with the CBPR process. Member checking was used to verify that the interpretation of the researcher matched the meaning that participants expressed through their photographs and interview transcript. Member checking occurred during and after the focus group to minimize bias in focus group dialogue and responses. Instead, the CAB was consulted following individual interviews to help authenticate that the researchers' interpretations coincided with the CAB's understanding of community issues and strengths. The focus group provided an additional chance for the researchers to triangulate their initial interpretations of the data through witnessing group interaction dynamics and testing interpretations.

### 2.3.Quantitative baseline survey

#### 2.3.1. Survey instrument

The World Health Organization (WHO) Alcohol Use Disorder Identification Test (AUDIT) was used to screen community members over the age of 18 for Alcohol-Use Disorders in Kuc. The AUDIT is a ten-question, Likert scale survey designed to gauge alcohol consumption and validated for use in a variety of cultural and geographic contexts [66]. Based on published guidelines, respondents are assigned an AUDIT score and then categorized to one of four levels of alcohol use: Low Risk (AUDIT Score 0–7), Hazardous drinking (AUDIT Score 8–15), Harmful Use (AUDIT Score 16–19), and Alcohol dependence (AUDIT Score 20–40).

#### 2.3.2. Sampling and analysis

Between September and October 2019, 327 AUDIT surveys were administered. At the start of the study, a household and individual community member list did not exist for Kuc, but a list of household heads was compiled for the purposes of this research through a special request to the Local Council 1 and Rwodi (local village leaders). The list contained a total of 446 households. The household list was digitized, and random numbers were generated in the statistical program R [67] to assign each household a number between 1 and 446. Beginning with household 1 on that list, all individuals 18 years and older were invited to participate until the minimum sample size was met. The sample size was based on available funding and time for the data collection; however, a minimum sample size was calculated of 309 people to achieve a 90% confidence level with a 4% margin of error. The AUDIT was administered by a trained research assistant using an Acholi translated AUDIT with the first author present to help with the calculation of alcohol units consumed each respondent.

Alcohol consumption was measured in alcohol units, defined as the equivalent of 10 grams of alcohol. For each consumption question, community members were asked about the quantity and type of alcohol they normally consume on a day when they consume alcohol. The Alcohol by Volume (ABV) for common alcoholic beverages consumed in Kuc was estimated based on the ABV denoted on the packaging for recorded alcohol, and through literature on locally brewed alcohol. Common alcohol types consumed within this region include Lujutu (cassava-liquor; 21% ABV [68]), beer (5%, via label), Kwete (sorghum alcohol; 4% ABV [69]), waraji (banana-liquor; 40% ABV via label), and wine (12% ABV [70,71]). Standard containers for alcohol consumption in Kuc were measured (mL) and presented to community members as guides for volumetric estimation. Standard containers included a glass soda bottle (300 mL), large plastic cup (450 mL), sachet (100 mL), small liquor bottle (250 mL), and a large liquor bottle (750 mL). The following formula was used to identify the number of alcohol units consumed by participants for each consumption question:

$$Units=volume\;(mL)*\frac{ABV}{1000}*0.789$$

Probability weights were applied to the sample to correct for sampling bias [72] based on population proportions available from the Uganda Bureau of Statistics' most recent census available at the Parish level, including proportion of the population by sex and age groupings of 18–30, 31–59, and 60+. Weighted proportions of Low-Risk, Hazardous, Harmful, and Alcohol-Dependent community members were calculated, along with weighted samples calculated for each of the AUDIT questions. Bivariate and multivariate logistic regression were then used to quantify statistically significant associations (p < 0.10) and to calculate unadjusted and adjusted odds ratios for the impact of sex and age group on the likelihood of an individual having an alcohol-use disorder (defined as an individual with a AUDIT score of ≥ 8).

### 2.4.Ethics

Written informed consent was obtained from each Photovoice participant and AUDIT respondent via a signature or thumb print of the participant, the signature of a witness, and the signature of the researcher obtaining the informed consent. Photovoice participants also consented to their photography being used to demonstrate concepts and results in publication, and received training on photography and ethics, which included information about how to ethically obtain verbal informed consent from any person of whom they wanted to take a photo. This consent of community members who would appear in photos taken by Photovoice participants was required. Participants were trained to respect the privacy of community members who did not want to be photographed. In the photography and training ethics session, situational training was used to help participants identify inappropriate photos or situations they should not capture. Identifying features of all subjects in photographs were blurred in photo-altering software prior to storage on a secure hard drive, and photographs on the digital camera were permanently deleted. This methodology is in keeping with Photovoice and the entire research protocol was approved by the University of Florida Institutional Review Board in the United States (#IRB201702129), Clarke International University Research Ethics Commission in Kampala, Uganda (#IHSU-REC/0088), and the Uganda National Council for Science and Technology (#SS4745).

## 3. Results

Four prominent themes emerged from the eight individual interviews and focus group discussion related to Photovoice: alcohol related issues, sanitation and compound cleanliness, water quality and access, and infrastructure. The four themes were initially generated in the individual interviews, and the focus group discussion provided valuable insight into agreement and disagreement among Photovoice participants, especially related to the spatial location of residence within Kuc. Selected quotations from participants and photographs are used to illustrate the themes identified in interviews. Participants felt alcohol-related issues were the most serious health issue facing Kuc. The three-hundred and thirty-seven AUDIT surveys confirmed the high levels of alcohol-use disorders in Kuc, especially among men.

### 3.1.Photovoice

#### 3.1.1. Alcohol related health impacts

Alcohol-use was a common theme and the most common discussion point across all photos. During individual interviews, every participant identified alcohol as the greatest health issue facing Kuc, which was confirmed during group discussion in the focus group. Concerns related to alcohol included both the direct consequences of consumption and tangential influences on health, including impacts on family life, planning for the future, living with HIV/AIDS, and sanitation and home-dwelling repair.

Alcohol has a profound impact on relationships and family dynamics in Kuc. As a result of excessive alcohol consumption, family separation, domestic violence, and the early death of family members due to excessive alcohol consumption weighs heavily on both domestic and community life.

*Husband and wife are getting separated because of alcohol…I lost one of my brothers due to alcohol. He used to drink so much that he started vomiting out blood. We stopped him from drinking alcohol, but he drunk until he breathed his last breath*.

                         -Participant #1, Individual Interview, Female

The relatively large amount of income directed towards alcohol in some families has also caused problems in Kuc and can also influence fighting between spouses and negatively impact children.

*Because the husband drinks all the money, the wife asked him to provide money to buy sauce, to which the husband says he doesn't have money. They began fighting, and the woman, because she was angry with the man, although she had money, she refused to pull out her money to buy sauce for the children. That means that domestic violence will always affect kids*.

                         -Participant #8, Individual Interview, Male

There was also a worry among participants about the impact alcohol has on families to function and plan for the future, noting alcohol’s contribution to the lack of sanitation facilities or poor compound upkeep.

*There are community members who drink, and they don't have any plan to make their compound better. The only plan that they have is to wake up in the morning and go and drink, without having to construct a bathroom, a toilet, or cleaning their compounds*.

                         -Participant #3, Individual Interview, Female

**Fig 1:**
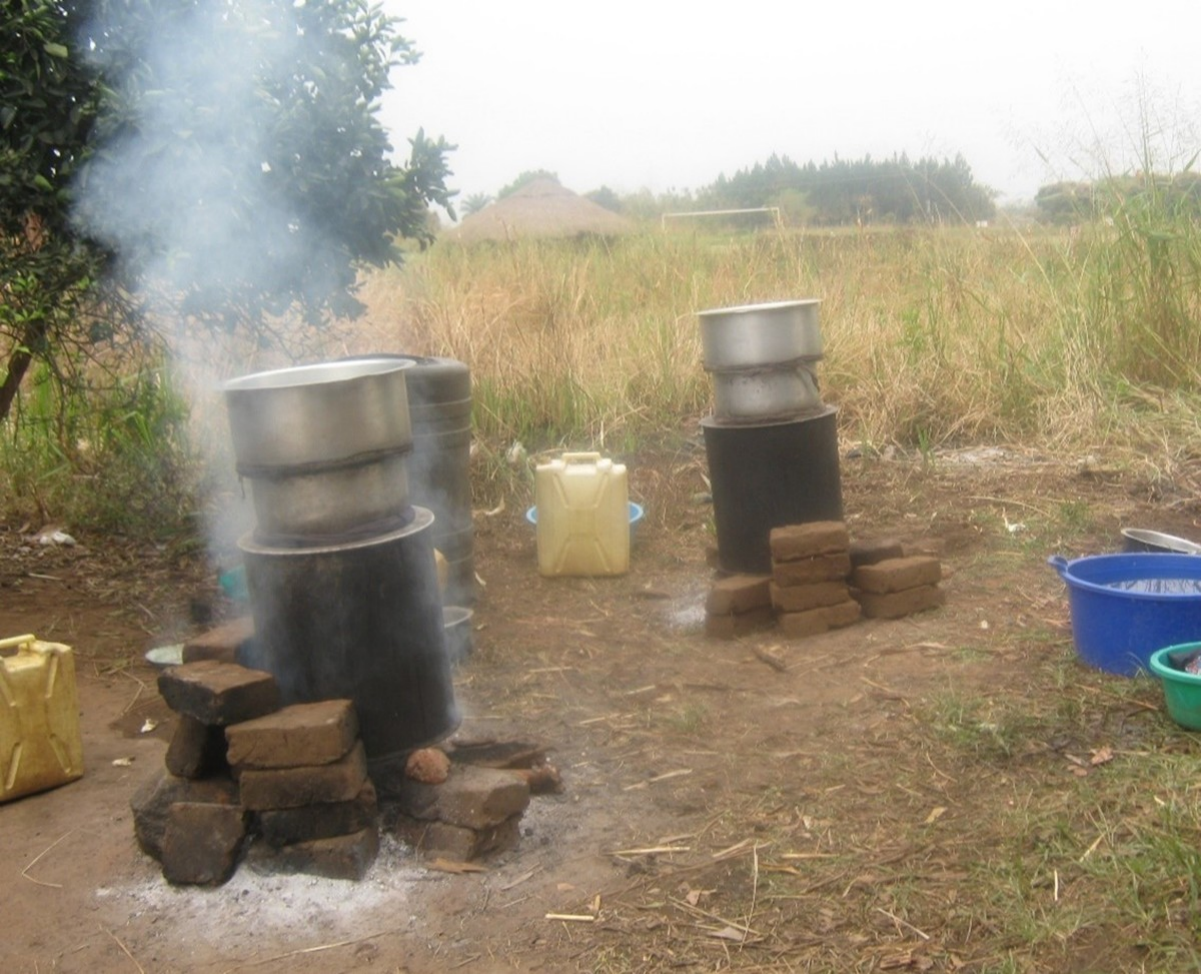
An example non-recorded alcohol being brewed in the village.

Even participants who were themselves brewers of the local alcohol consumed by community members were worried about alcohol's negative impacts (Fig 1). Community-brewed alcohol is termed "nonrecorded alcohol", since it is not measured or monitored due to its exclusion from data on tax, sales, and production. The money from brewing is used to fund school fees and other household responsibilities, but the wide availability of cheap, locally brewed alcohol means it is readily available in Kuc.

Participants described their displacement during the war in IDP camps as having changed drinking habits within Kuc, with community members drinking higher-alcohol content liquor during and after living in the camps. Additionally, community members began drinking at higher rates earlier in the day.

*Before people came into the camp, the drinking was mild. The type of alcohol that the elders used to drink was mild. It was not the type of alcohol that would make you crazy. It was an organized type of drinking that was usually done after farming a large piece of a garden. It was a kind of drinking that was in appreciation of hard work from a group of people farming together*.

                         -Participant #6, Focus Group, Female

Participants also describe alcohol as having contributed to an increase in HIV prevalence in the community through increased sexual promiscuity and risky sexual interactions, highlighting an interaction between what is traditionally considered a non-infectious disease (alcohol-use disorder) and an infectious disease (HIV/AIDS).

*Alcohol makes you think you are great and can do everything. I blame the rate of increased HIV on alcoholism…It is not only a problem within Kuc, it is a problem within Uganda*.

                         -Participant #8, Focus Group, Male

Upon inquiring about solutions to alcohol related issues in Kuc, participants generally did not favor prohibition, but rather indicated a desire to limit the timeframe when alcohol is legally consumed, or to implement more punitive solutions, such as arrest for public intoxication.

#### 3.1.2. Water quality

Water quality and access were also presented as serious issues in Kuc, but varied based on the geographic location where individuals lived within Kuc. During the focus group discussions, participants disagreed about the extent of water quality issues, however, the participants who did experience issues in water quality and access often experience severe gastrointestinal illness, as a result. Participants agreed in the focus group that water quality and access varied based on the participant’s residential location within the village.

The main issues regarding water quality stem from broken boreholes and multi-use water sources. Boreholes break when the pump is mis-used, parts are stolen for agricultural activities, or poor-quality repairs are made. Participants speculated that mechanics deliberately repair wells poorly to ensure profit from future repairs. In such cases, even when a community has a borehole in their environs, lack of proper maintenance and theft means community members must trek further to another borehole or obtain water from streams and other open waterbodies. When safe water collection points are not available, water is collected in streams where other people are simultaneously bathing, washing clothing, commuting through the stream, and where animals use the stream to cool down or drink water.

*In that picture, those girls standing right there are fetching that water for drinking…There are children bathing, and this very man is moving across carrying his bicycle. I found it inappropriate for people to be drinking that water. It might cause them disease*.

                         -Participant #1, Individual Interview, Female

Despite obtaining water from collection points where water quality is poor, participants also indicated that water is not properly boiled after collection. In such cases, community members are either not aware of the health benefits of boiling water or prefer the taste of non-boiled water. Similarly, water storage containers are often not properly cleaned, resulting in the contamination of boiled water.

Solutions to water quality and access issues solutions the community members put forward included building new boreholes where there is a lack of clean water, employing a trusted caretaker to ensure proper use and maintenance of boreholes, and creating a bylaw to govern proper use of boreholes through a fine for those who misuse or break boreholes.

#### 3.1.3. Sanitation and compound maintenance

**Fig 2:**
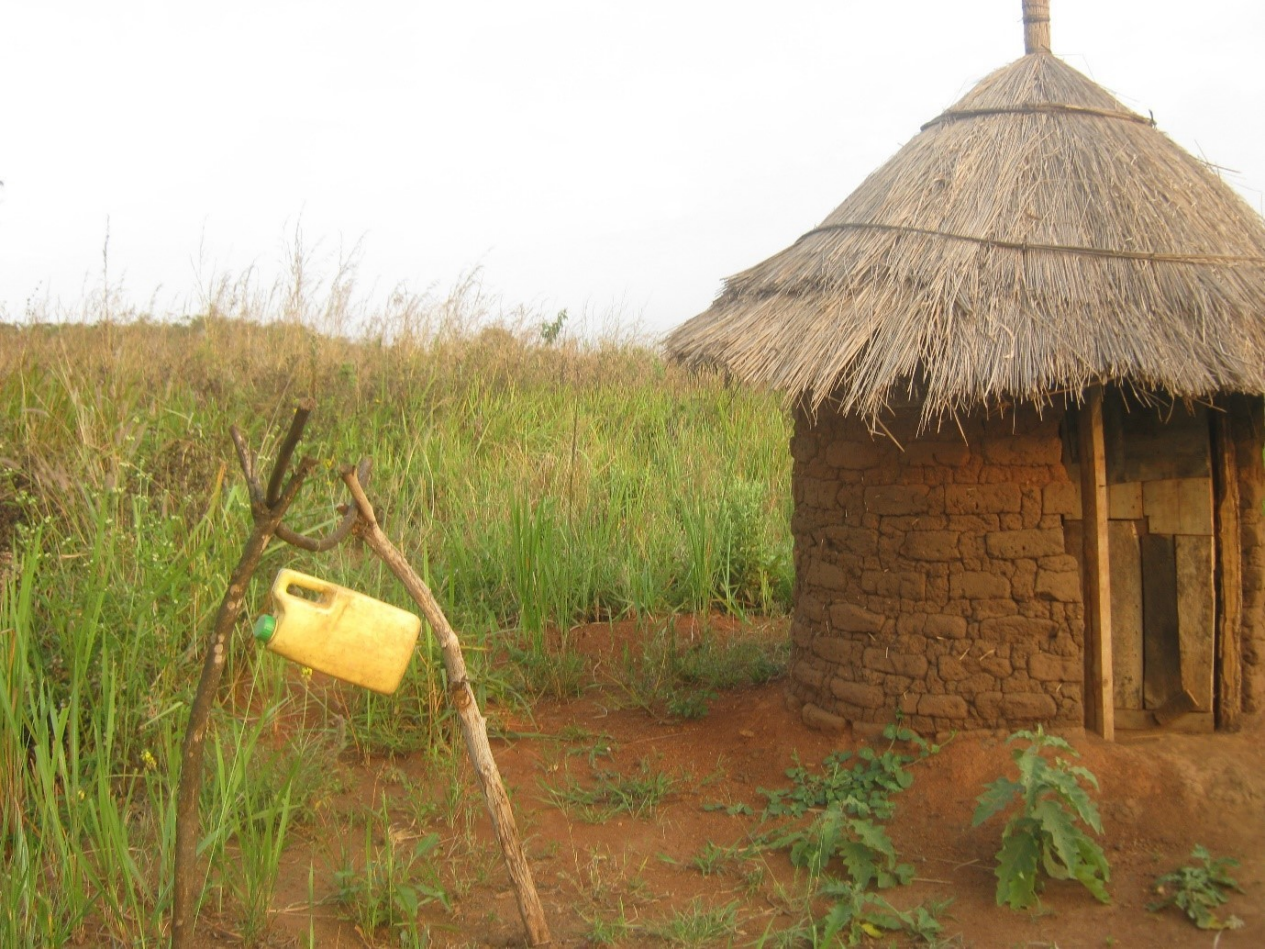
An example of a proper sanitation set up, with a well-constructed latrine and hand washing station.

Participants felt sanitation and proper hygiene to be important for the maintenance and promotion of good health in Kuc. Participants focused much of their discussions of sanitation and compound maintenance on proper sanitation structures and tools in family compounds, including the importance of a well-built latrine to control fecal waste (Fig 2).

*If you have a latrine, it's easy to control the wastes around the home, especially the fecal waste. Usually, when you have kids around and you don't have a latrine, they have a tendency to go and pass stool everywhere and it may be hard to control the flies that come around to the feces. Flies will come on the feces and again drop in your drinking water, drop in your food. During the mango seasons, the children will pick the mangos even nearest to the feces, and that might cause fever to the children*.

                         -Participant #2, Individual Interview, Male

In addition to latrines, participants stressed the importance of other sanitation items, including dish drying racks and rubbish pits to prevent the spread of disease by elevating eating utensils and allowing dishes to dry to kill waterborne pathogens. In addition, slashing of grass near the compound was considered vital to reducing the chance of having a snake bite. One of the participants recently lost an infant family member due to a snake bite, where the snake was hiding in the long grass near a latrine, highlighting the impact of compound maintenance on family health and safety.

Participants commented that sanitation issues could best be addressed through community sensitization, where community experts teach other community members on proper latrine, dish drying rack, hand washing station and rubbish pit construction and maintenance. Additionally, they believe continued and increased enforcement of current bylaws requiring sanitation items to be necessary.

#### 3.1.4. Infrastructure

**Fig 3:**
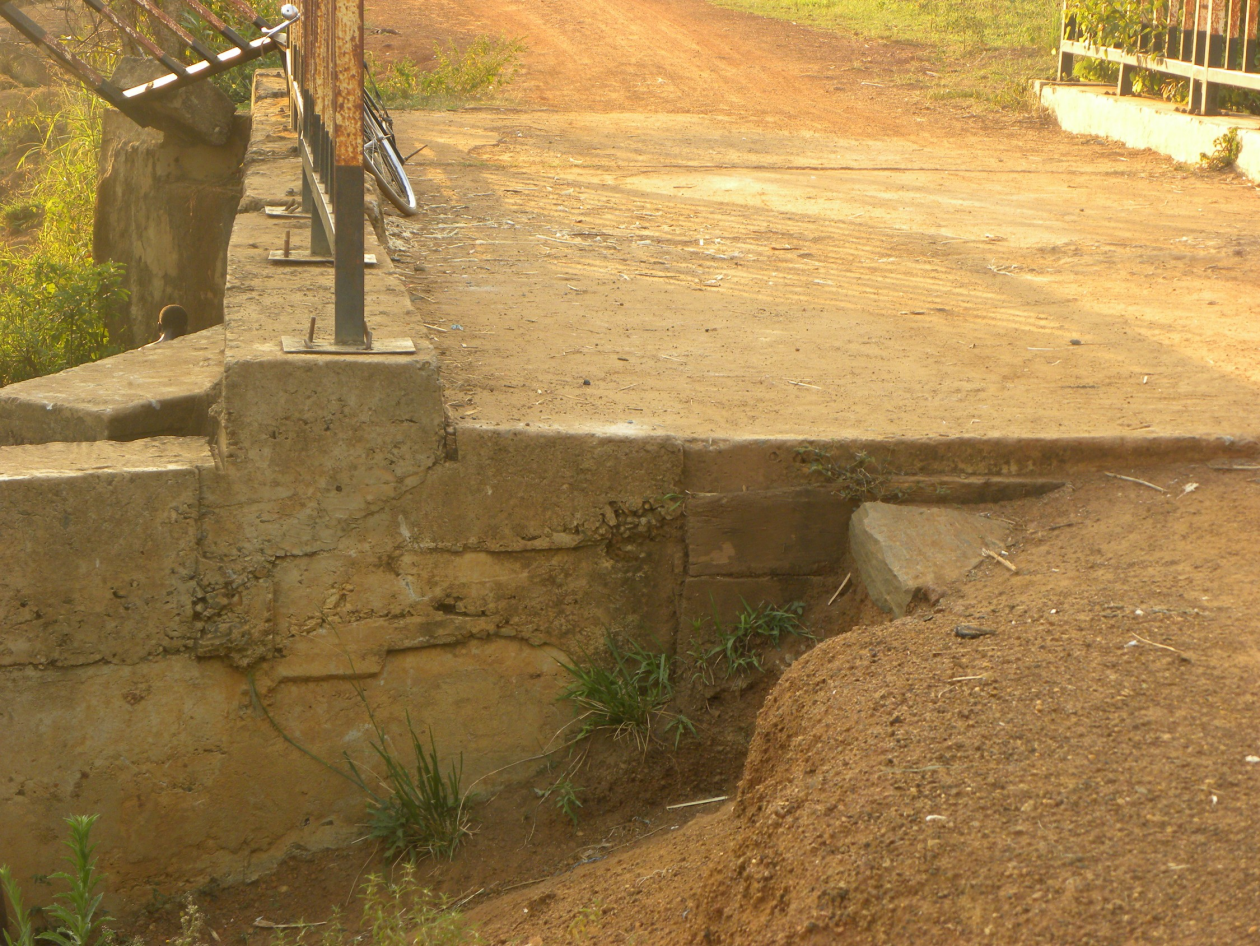
An example of deteriorating infrastructure. In this example, the respondent detailed numerous accidents that have occurred due to this degraded bridge on a busy road.

Poor infrastructure also emerged as a common theme among participants responses, although this varied by the location of residence of respondents, largely driven by inconsistent road quality. Disagreement among participants in the focus group regarding the quality of roads and the significance of this issue for community health was observed. Individuals who lived near the graded main road expressed satisfaction with road quality. However, individuals who lived in parts of the village away from the road were adamant about its poor quality and the impact of that reality on their life. Poor road access to the town center results in individuals taking dangerous measures to shorten their trip, such as crossing a flooded swamp or river to get home and can make it more difficult for them to access health and education centers. Infrastructure is also in desperate need of repair, with dangerous roads for motorcycles to pass, leading to fatal accidents (Fig 3). Potential solutions to fix infrastructure related issues were political in nature. Participants expressed the need for community members to advocate for the repair of roads by pressuring leaders.

*This is a very big bridge…but this side has a big hole that is a danger to people's lives…There were two motorbikes that were coming from Gulu Town that fell there…They were taken to the hospital. This is a great hazard to our lives*.

                         -Participant #6, Individual Interview, Female

#### 3.1.5. Community strengths

In addition to highlighting the major health issues in their community, participants were asked to identify what they saw as strengths within their community with regard to improving health. Participants provided examples of community efforts to mitigate negative health influences in Kuc, including community-based interventions to curb negative health behavior, and efforts of community members and village health workers to help vulnerable individuals maintain proper sanitation infrastructure within their compounds. Community institutions, such as schools, also play a vital role in influencing parent’s behavior, including saving money for school fees and encouraging them to spend less money on alcohol and more on their children.

*I want to commend the work of the school in reducing the consumption of alcohol. Whenever there is a school meeting…there are parents who come drunk. Not only coming drunk, there are other parents who could not clear their children's school fees for the term. And, when they come for school meetings, the leadership of the school disciplines the…There are times when each parent drinks 1000 shillings each day and cannot afford to pay an examination fee of 5000. Such examples were given to those parents who drink so much, and they were able to save 1000 shilling to pay for their children's examination fee of 5000 shillings*.

                         -Participant #8, Individual Interview, Male

Community members and community groups were highly active in addressing issues related to sanitation and household maintenance. For instance, Non-Governmental Organizations (NGOs) periodically train certain community members on proper sanitation habits, and the trained community members become community advocates using their new expertise.

*Within this community, we have elected lead persons to head the campaigns of having a clean home. The lead persons are going to receive training, and they are the people who are going to come back to the community and teach the community how to build the latrines*.

                         -Participant #6, Individual Interview, Female

Village Savings and Loans Associations (VSLA) have been adapted to help elderly and disabled community members ensure their compounds have proper latrines, drying racks, and that the compounds are properly cleaned every month. Members also help each other clean their compounds and build important sanitation items. Village Savings and Loans Associations are used to buy construction items in bulk, such as iron sheets, to save money for the community as a whole. Punitive measures are used to ensure members are following village health codes, including withholding savings payouts.

*Not only is [the savings group] improving the cleanliness of the families, but it is also making women save more. Last year, they came into my compound, which they do every Thursday, and make sure your compound is cleaned. Everything is cleaned, from the bathroom, the house, the compound, everything is left cleaned. And they are also saving to buy tin roofs, like iron sheets, to build more permanent houses…In our savings group, when we come to your home, and you have only one hut, meaning you don't have a different hut for boys and girls, even if you were to be number one on the list to get your savings, we shall put your name at the back, until we find that everything is in order. We want a boy from twelve years to have their own hut, and all the other small things, like the bathroom, the toilet, the rubbish bin, things for putting plates, should be on your compound*.

                         -Participant #1, Individual Interview, Female

The sub-county and VHT are also actively involved in sanitation. Government officials visit village compounds to inspect for proper sanitation systems. Occasionally, gifts are given to residents who have exceptionally clean and well-maintained compounds, while punitive measures are enforced against those who do not. When someone does not have a latrine, the sub-county will build a latrine, but will confiscate an asset, such as livestock, to pay for building expenses. It is through these community-based measures that members of Kuc hold each other accountable to creating a healthier and safer community.

### 3.2. Quantitative baseline survey

A total of 327 individuals completed the AUDIT survey, including 187 females and 140 male participants. The age of participants ranged from the age of 18–87 (mean 39; standard deviation (SD) 16; median 37). Internal consistency was excellent, with a Cronbach's alpha of 0.93. A description of participants AUDIT classifications is presented in Table 3. Most community members (76.45%) were abstinent or low-risk, while 4.28% were classified as Harmful Users, 8.26% as Hazardous Users, and 11.01% as Alcohol Dependent. Men were significantly more likely to be classified with an alcohol-use disorder than women (Table 4; OR = 7.234; p < 0.001).

**Table 3:** Prevalence (%) of alcohol-use disorders in Kuc, based on the Alcohol Use Disorder Identification Test (AUDIT).

Demographic Segment	Low Risk or Abstinence (90% C.I)	Harmful Use (90% C.I)	Hazardous Use (90% C.I)	Alcohol Dependence (90% C.I)
**Total**	76.45% (72.39–80.09)	4.28% (2.78–6.53)	8.26% (6.08–11.12)	11.01% (8.48–14.18)
**Sex**				
Women	91.13% (86.80–94.13)	1.55% (0.58–4.07)	4.17% (2.27–7.54)	3.16% (1.57–6.25)
Men	58.67% (52.22–64.84)	7.30% (4.60–11.41)	13.36% (9.56–18.37)	20.66% (15.93–26.35)
**Age Group**				
18–30	80.07% (73.96–85.04)	3.13% (1.46–6.59)	7.50% (4.59–12.03)	9.30% (5.99–14.15)
30–59	71.73% (65.34–77.35)	4.93% (2.74–8.72)	9.19% (6.00–13.83)	14.15% (10.10–19.47)
60+	69.98% (56.77–80.54)	6.89% (2.62–16.88)	11.30% (5.32–22.43)	11.83% (5.66–23.06)
**Age Group and Sex**				
*Women*				
18–30	94.74% (88.49–97.68)	1.32% (0.28–5.94)	2.63% (0.84–7.91)	1.32% (0.28–5.94)
30–59	89.41% (82.17–93.93)	1.18% (0.24–5.48)	4.71% (2.04–10.50)	4.71% (2.04–10.50)
60+	84.62% (66.98–93.72)	3.85% (0.71–18.37)	7.69% (2.20–23.62)	3.85% (0.71–18.37)
*Men*				
18–30	65.00% (55.14–73.73)	5.00% (2.13–11.27)	12.50% (7.33–20.51)	17.50% (11.24–26.22)
30–59	53.75% (44.28–62.96)	8.75% (4.71–15.68)	13.75% (8.45–21.60)	23.75% (16.65–32.70)
60+	55.00% (36.71–72.03)	10.00% (3.26–26.80)	15.00% (5.99–32.81)	20.00% (9.09–38.46)

**Table 4:** Odds Ratio (OR), 90% Confidence Intervals (CI), and P-values (P) for bivariate and multivariate impact of sex and age on alcohol-use disorders.

Characteristic	Bivariate Analysis			Multivariate Analysis		
Sex	**OR**	**90% CI**	**P**	**OR**	**90% CI**	**P**
Female	Ref			Ref		
Male	7.234	4.299–12.175	< .001	7.396	4.379–12.492	< .001
Age	**OR**	**90% CI**	**P**	**OR**	**90% CI**	**P**
18–30	Ref			Ref		
31–59	1.58	1.001–2.505	0.099	1.695	1.034–2.779	0.079
60+	1.723	0.875–3.396	0.187	1.882	0.899–3.941	0.159

According to the responses on individual questions of the AUDIT, daily drinking patterns and quantity of alcohol consumed, the impact of alcohol on social interactions and daily responsibilities, and the prevalence of physical harm from alcohol varied by sex and age-group (Table 5). Men had a higher prevalence of high-risk drinking habits, with 22.96% of men reportedly consuming more than six drinks on a typical drinking day compared to just 3.6% of women. Men also had a higher prevalence of the consumption of six or more drinks daily with 16.81%, compared to 0.45% of women, and 39.02% of men report heavy episodic drinking (defined as consuming six or more drinks at least once a month), compared to 8.31% of women.

**Table 5:** Responses by demographic segment for the Alcohol Use Disorder Identification Test (AUDIT).

		Demographic Segment											
					All Population in Age Group			Women in Age Group			Men in Age Group		
Question	Answer	Total	Women	Men	18–30	31–59	60+	18–30	31–59	60+	18–30	31–59	60+
**1. How often do you have a drink containing alcohol?**													
	Never	66.78%	83.75%	49.43%	69.34%	65.69%	61.67%	88.16%	82.35%	73.08%	50.00%	48.75%	50.00%
	Monthly or Less	7.58%	6.50%	8.67%	11.40%	4.83%	4.42%	7.89%	5.88%	3.85%	15.00%	3.75%	5.00%
	2–4 times a month	8.80%	5.57%	11.15%	5.60%	9.14%	15.19%	1.32%	7.06%	15.38%	10.00%	11.25%	15.00%
	2–3 times a week	8.33%	2.08%	15.09%	6.83%	9.86%	9.36%	1.32%	2.35%	3.85%	12.50%	17.50%	15.00%
	4 or more times a week	8.51%	2.08%	15.66%	6.83%	10.48%	9.36%	1.32%	2.35%	3.85%	12.50%	18.75%	15.00%
**2. How many drinks containing alcohol do you have on a typical day when you are drinking?**													
	0	66.78%	83.75%	49.43%	69.34%	65.69%	61.67%	88.16%	82.35%	73.08%	50.00%	48.75%	50.00%
	1 or 2	3.83%	3.81%	3.85%	3.80%	4.21%	2.47%	2.63%	5.88%	0.00%	5.00%	2.50%	5.00%
	3 or 4	6.35%	3.99%	8.77%	7.50%	3.05%	15.19%	2.63%	2.35%	15.38%	12.50%	3.75%	15.00%
	5 or 6	9.86%	4.84%	14.99%	10.07%	11.05%	4.42%	5.26%	4.71%	3.85%	15.00%	17.50%	5.00%
	7, 8, or 9	3.48%	1.43%	5.58%	2.47%	3.07%	8.83%	0.00%	1.18%	7.69%	5.00%	5.00%	10.00%
	10 or more	9.70%	2.17%	17.38%	6.83%	12.94%	7.41%	1.32%	3.53%	0.00%	12.50%	22.50%	15.00%
**3. How often do you have six or more drinks on one occasion?**													
	Never	75.12%	91.13%	58.77%	78.84%	72.35%	72.45%	94.74%	89.41%	84.62%	62.50%	55.00%	60.00%
	Less than monthly	1.38%	0.56%	2.22%	1.90%	0.62%	2.47%	1.32%	0.00%	0.00%	2.50%	1.25%	5.00%
	Monthly	4.80%	3.09%	6.54%	6.27%	3.67%	3.89%	2.63%	2.35%	7.69%	10.00%	5.00%	0.00%
	Weekly	10.16%	4.77%	15.67%	6.83%	12.83%	11.83%	1.32%	8.24%	3.85%	15.50%	17.50%	20.00%
	Daily or almost daily	8.54%	0.45%	16.81%	6.17%	10.54%	9.36%	0.00%	0.00%	3.85%	15.50%	21.25%	15.00%
**4. How often during the last year have you found that you were not able to stop drinking once you had started?**													
	Never	86.25%	95.74%	76.55%	88.80%	84.02%	85.70%	97.37%	94.12%	96.15%	80.00%	73.75%	75.00%
	Less than monthly	6.29%	2.11%	10.57%	6.27%	6.17%	6.89%	2.63%	1.18%	3.85%	10.00%	11.25%	10.00%
	Monthly	0.57%	0.00%	1.15%	0.00%	0.62%	2.47%	0.00%	0.00%	0.00%	0.00%	1.25%	5.00%
	Weekly	1.90%	2.15%	1.64%	1.23%	2.99%	0.00%	0.00%	4.71%	0.00%	2.50%	1.25%	0.00%
	Daily or almost daily	4.99%	0.00%	10.09%	3.70%	6.20%	4.94%	0.00%	0.00%	0.00%	7.50%	12.50%	10.00%
**5. How often during the last year have you failed to do what was normally expected from you because of drinking**													
	Never	80.94%	92.11%	69.52%	81.30%	80.38%	81.81%	94.74%	90.59%	88.46%	67.50%	70.00%	75.00%
	Less than monthly	11.38%	5.29%	17.60%	13.77%	9.81%	8.83%	5.26%	4.71%	7.69%	22.50%	15.00%	10.00%
	Monthly	0.57%	0.00%	1.15%	0.00%	1.24%	0.00%	0.00%	0.00%	0.00%	0.00%	2.50%	0.00%
	Weekly	3.29%	1.61%	5.00%	2.47%	4.26%	2.47%	0.00%	3.53%	0.00%	5.00%	5.00%	5.00%
	Daily or almost daily	3.83%	0.98%	6.73%	2.47%	4.31%	6.89%	0.00%	1.18%	3.85%	5.00%	7.50%	10.00%
**6. How often during the last year have you needed a first drink in the morning to get yourself going after a heavy drinking session?**													
	Never	91.74%	96.73%	86.64%	92.50%	90.19%	95.06%	97.37%	95.29%	100.00%	87.50%	85.00%	90.00%
	Less than monthly	3.55%	1.10%	6.06%	4.37%	3.69%	0.00%	1.32%	1.18%	0.00%	7.50%	6.25%	0.00%
	Monthly	0.00%	0.00%	0.00%	0.00%	0.00%	0.00%	0.00%	0.00%	0.00%	0.00%	0.00%	0.00%
	Weekly	1.68%	1.64%	1.72%	0.67%	3.05%	0.00%	1.32%	2.35%	0.00%	0.00%	3.75%	0.00%
	Daily or almost daily	3.03%	0.54%	5.58%	2.47%	3.07%	4.94%	0.00%	1.18%	0.00%	5.00%	5.00%	10.00%
**7. How often during the last year have you had a feeling of guilt or remorse after drinking?**													
	Never	83.49%	95.27%	71.46%	86.90%	81.51%	78.81%	96.05%	95.29%	92.31%	77.50%	67.50%	65.00%
	Less than monthly	8.26%	3.74%	12.88%	6.93%	8.60%	11.83%	3.95%	3.53%	3.85%	10.00%	13.75%	20.00%
	Monthly	2.46%	45.00%	4.51%	1.23%	3.10%	4.42%	0.00%	0.00%	3.85%	2.50%	6.25%	5.00%
	Weekly	3.60%	0.54%	6.72%	2.47%	4.93%	2.47%	0.00%	1.18%	0.00%	5.00%	8.75%	5.00%
	Daily or almost daily	2.19%	0.00%	4.43%	2.47%	1.86%	2.47%	0.00%	0.00%	0.00%	5.00%	3.75%	5.00%
**8. How often during the last year have you been unable to remember what happened the night before because you had been drinking?**													
	Never	84.74%	92.76%	76.54%	88.13%	83.45%	77.39%	96.05%	91.76%	84.62%	80.00%	75.00%	70.00%
	Less than monthly	6.22%	3.16%	9.35%	6.83%	4.85%	9.36%	1.32%	4.71%	3.85%	12.50%	5.00%	15.00%
	Monthly	3.01%	2.02%	4.02%	1.33%	3.72%	6.36%	2.63%	0.00%	7.69%	0.00%	7.50%	5.00%
	Weekly	4.08%	2.06%	6.15%	2.47%	4.88%	6.89%	0.00%	3.53%	3.85%	5.00%	6.25%	10.00%
	Daily or almost daily	1.94%	0.00%	3.93%	1.23%	3.10%	0.00%	0.00%	0.00%	0.00%	2.50%	6.25%	0.00%
**9. Have you or someone else been injured as a result of your drinking?**													
	No	78.98%	91.53%	66.15%	78.74%	79.73%	76.87%	92.11%	91.76%	88.46%	65.00%	67.50%	65.00%
	Yes, but not in the last year	8.88%	5.71%	12.11%	9.40%	9.16%	5.84%	3.95%	5.88%	11.54%	15.00%	12.50%	0.00%
	Yes, during the last year	12.15%	2.76%	21.74%	11.87%	11.10%	17.30%	3.95%	2.35%	0.00%	20.00%	20.00%	35.00%
**10. Has a relative or friend or doctor or another health worker been concerned about your drinking or suggested you cut down?**													
	No	70.63%	88.90%	51.95%	75.60%	66.80%	67.51%	90.79%	88.24%	84.62%	60.00%	45.00%	50.00%
	Yes, but not in the last year	8.20%	4.28%	12.21%	8.17%	8.57%	6.89%	3.95%	4.71%	3.85%	12.50%	12.50%	10.00%
	Yes, during the last year	21.17%	6.81%	35.84%	16.23%	24.63%	25.60%	5.26%	7.06%	11.54%	27.50%	42.50%	40.00%

## 4. Discussion

In this study, Photovoice was used to engage community members in Kuc, Uganda in bottom-up research agenda setting. The results detailed in this paper, including the triangulation of community identified need and disease burden, have implications for the design of research and interventions, highlighting the importance of locally derived priorities and data when designing and implementing community-level interventions and programs. By giving participants cameras to document their perceptions of health using Photovoice, Kuc community members selected alcohol-use disorders as the topic for future public health research and intervention. A survey was then conducted, the results of the which indicated 23.55% of Kuc adults had an alcohol-use disorder, 16.45 percentage points higher than WHO estimates for Uganda [73], thus verifying the results of Photovoice through triangulation. The importance of the results of this study are thus two-fold: the *process* of collecting data to identify community-based priorities for research and *results* of that research process.

The research process and approach taken in this endeavor underscores the importance of community-based approaches and demonstrates one (of, no doubt, many) bottom-up approach. Importantly, the research illustrates the effectiveness of using Photovoice as a method to elicit community priorities for research and practice, particularly when used in tandem with other methods that allow for triangulation and validation/invalidation of those findings. This process led to identification of alcohol-use disorders as a priority intervention. That alcohol-use disorders, which are a Non-Communicable Disease (NCD), were identified by the community as their highest priority highlights the importance of community engagement in research priority setting, as researchers and practitioners navigate the simultaneous burden of NCDs and infectious disease in LMICs. While NCDs accounted for 73% of mortality and 62% of all global Disability-Adjusted Life Years (DALYs) in 2017 and are continuing to increase rapidly, Development Assistance for Health only allocated 1.78% of the total global budget to all NCDs [74,75]. This divide between funding, disease burden, and, by extension, action, in public health research in LMICs could inhibit sensitivity to local priorities. Working with communities to identify priorities may help overcome this divide.

The results from photovoice and the follow up survey point to alcohol-use disordersas a major health concern for the people of Kuc. Prior studies indicate that Uganda has one of the highest alcohol consumption rates in the world [76–78]. Previous studies using the AUDIT in northern Uganda found a prevalence of alcohol use disorders among 16.2% of adult IDPs in Gulu and Amuru districts in 2006 [79], 21.5% in rural communities in northern Uganda in 2010 [71], and 4.3% among communities in Gulu, Amuru, and Nwoya districts in 2012 [80]. Results presented here show higher levels and more severe patterns of alcohol-use disorders (including alcohol dependence) than all three prior analyses. Participants expressed the negative impacts of alcohol on households, families, and the community-at-large. While there remains limited literature on the impacts of alcohol-use disorders in Uganda [70,81,82], available research has shown the increased vulnerability of excess alcohol-consumption for individuals of lower socioeconomic and educational backgrounds due to a reduction in social and environmental buffers from harm, and a higher proportion of income directed towards the consumption of alcohol [77,83]. Considering alcohol is known to be fully or partially responsible for approximately 230 diseases and injuries globally [84], the burden of alcohol on health and the efficacy of community-based and approved solutions for alcohol-use disorders in Kuc merit investigation and attention.

It is important to note that, in addition to alcohol-use disorders, Photovoice participants identified two other major health issues in Kuc: water quality and sanitation and poor infrastructure. Water quality and sanitation emerge as prominent themes in other Photovoice studies of health in East Africa [57,58,85,86]. In rural Uganda, only 66% of villages have access to a source of safe water and 36.5% of households have hand washing facilities with both soap and water [87], and enteric infections account for 5.52% of deaths and 5.16% of DALYs in Uganda [74]. Community solutions proposed by Photovoice participants, such as employing a care-taker to ensure proper-use and maintenance of drinking water access points, matches best-practices outlined by the World Bank, whereby strong local management is correlated with proper maintenance and protection of water resources [88]. Poor infrastructure, cited as a barrier to education and economic opportunity in Kuc, is a well-documented constraint to health. Road injuries accounting for 2.51% of all deaths and 2.00% of DALYs in Uganda [74]. While an improved road network can directly influence health by connecting communities to important health services [89,90], it is also an important economic driver for rural communities, increasing economic welfare, addressing some of the social-determinants of health [91,92] and many of the SDGs [93–96]. Although road networks in Uganda have improved drastically over the past decade [97], Ugandan residents remain overwhelmingly frustrated and unsatisfied with the quality of roads and infrastructure throughout the country [98].

The bottom-up agenda setting process documented in this paper highlights the ethical and practical benefits of partnering with target communities in public health programming. These results highlight the tension between what Pratt [7] calls ‘macro level’ decisions and priority setting from external research funders and donors, and priorities of target communities. The Kuc community selected the underfunded and understudied health issue of alcohol-use disorders [99] to guide future study and public health action in the community. By augmenting current donor-driven agenda setting with a bottom-up agenda setting approach like the one outlined in this paper, the divide between local realities and global priorities could be further bridged.

### Study strengths and limitations

Compared to past Photovoice studies conducted in this region, this analysis triangulates findings using quantitative data to verify results and strengthen Photovoice conclusions. This paper found stark differences in alcohol-use disorders compared to prior prevalence studies within northern Uganda [71,79,80]. The higher level of alcohol-use disorders in this paper are likely due to time since data collection, the different communities and population types included in the study, and problems limiting the comparability of studies using the AUDIT [100], namely the cutoff point used to evaluate probable alcohol-use disorders and the definition of a standard unit of alcohol. Although the authors were able to standardize the cutoff points for comparison between these three studies, most of the three prior studies used unclear or imprecise alcohol unit definitions. For instance, Blair et al. [80] considered a sachet of Waragi (100 mL at 40% ABV) and a single beer (generally 500 mL at 5% ABV) to be one unit of alcohol, while not specifying the local alcohol ABV percentages used in their calculation. Using the formula outlined in this paper's methods section, a sachet of Waragi would equate to 3 units, while a 500 mL beer would equate to 2 units, likely resulting in a significant under-reporting of alcohol-use disorders in the Blair et al. [80] paper. Similarly, Roberts et al. [79] used the typologies of "1 glass of waragi, one bottle or glass of local brew or one bottle of beer" which also was likely an underestimation. Ertl et al. [71] used the definition of one unit equal to 13g of pure ethanol and identified ABV of recorded alcohol, but appear to have excluded calculations of unrecorded alcohol, which is most prevalent in rural communities. Thus, this study represents the first in the region to clearly state ABV for common alcoholic drinks consumed in this region, including unrecorded alcohol.

The results must be viewed against potential limitations of this study. While the overall goal was to utilize Photovoice to gain rich data from few participants, caution must be used when extrapolating the results of this study outside of the village of Kuc to other areas of Uganda or East Africa. Rather, this study helps to illuminate community perspectives and priorities within the realm of health and highlights how Kuc community members view health in line with a social-ecological lens [101,102]. Sampling for AUDIT was completed using a random-clustered approach based on all individuals in a household that are 18 and older. If specific demographics were absent from the household due to working or travelling away from home, the representativeness of the sample could be impacted. Sampling weights were used as an attempt to correct for over and under sampling. Additionally, it has been suggested that AUDIT cutoff points be reduced in LMICs due to the regressive impacts of alcohol in poorer households [66,80,100]. In this analysis, the researchers refrained from lowering the AUDIT cutoff, as there has not been a verified psychometric analysis of the AUDIT instrument in this study location. Lowering the AUDIT cutoff would increase the sensitivity of the instrument. Thus, these numbers could be a conservative estimate of alcohol-use disorders in Kuc. The researchers suggest a psychometric analysis of the AUDIT be completed in northern Uganda, specifically, and Uganda, in general, to aid researchers in better understanding the likely burden of alcohol-use disorders within this region. Finally, recall bias should be considered as a limitation when interpreting the results of the AUDIT due to the survey assessing average participant alcohol consumption patterns over the twelve-month period leading up to the survey date.

## 5. Conclusions

This study used Photovoice as a research tool to identify and include a community in research-agenda setting and to better understand community perceptions regarding community health issues and strengths in Kuc, Uganda. The results identified alcohol as the main issue of community concern. A quantitative, cross-sectional survey using AUDIT was conducted to triangulate high levels of alcohol-use disorders within the Kuc community. The community identified issue of alcohol-use disorders will be the topic in the long-term CBPR inspired project. The project goal is the development, implementation, and evaluation of a community-driven public health program that builds on community strengths to address alcohol-use disorders in Kuc. At the time of publication of this article, focus groups have been conducted with community members and VHTs on community opinions and perceptions of potential alcohol-use disorder, evidence-based public health programs to implement in Kuc that build off community strengths. As suggested by the results of this study, if public health is to maintain sensitivity to local realities, including local perspectives in research and practice, bottom-up agenda setting is a must to ensure health issues of community importance are addressed.

## Supporting information

S1 Data Deidentified AUDIT dataset. (CSV)
